# The Effect of Age and NT-proBNP on the Association of Central Obesity with 6-Years Cardiovascular Mortality of Middle-Aged and Elderly Diabetic People: The Population-Based Casale Monferrato Study

**DOI:** 10.1371/journal.pone.0096076

**Published:** 2014-05-01

**Authors:** Graziella Bruno, Federica Barutta, Andrea Landi, Paolo Cavallo Perin, Gabriella Gruden

**Affiliations:** Dept. of Medical Sciences, University of Turin, Turin, Italy; "Mario Negri" Institute for Pharmacological Research, Italy

## Abstract

**Background:**

Among people with type 2 diabetes the relationship between central obesity and cardiovascular mortality has not been definitely assessed. Moreover, NT-proBNP is negatively associated with central obesity, but no study has examined their combined effect on survival. We have examined these issues in a well-characterized population-based cohort.

**Methods and Findings:**

Survival data of 2272 diabetic people recruited in 2000 who had no other chronic disease have been updated to 31 December 2006. NT-proBNP was measured in a subgroup of 1690 patients. Cox proportional hazards modeling was employed to estimate the independent associations between cardiovascular and all-cause mortality and waist circumference. Mean age was 67.9 years, 49.3% were men. Both age and NT-proBNP were negatively correlated with waist circumference (r = −0.11, p<0.001 and r = −0.07, p = 0.002). Out of 2272 subjects, 520 deaths (221 for CV mortality) occurred during a median follow-up of 5.4 years. Central obesity was not associated with CV mortality (hazard ratio, HR, adjusted for age, sex, diabetes duration, 1.14, 95% CI 0.86–1.52). NTproBNP was a negative confounder and age a strong modifier of this relationship (p for interaction<0.001): age<70 years, fully adjusted model HR = 3.52 (1.17–10.57) and age ≥70 years, HR = 0.80 (0.46–1.40). Respective HRs for all-cause mortality were 1.86 (1.03–3.32) and 0.73 (0.51–1.04).

**Conclusions:**

In diabetic people aged 70 years and lower, central obesity was independently associated with increased cardiovascular mortality, independently of the negative effect of NT-proBNP. In contrast, no effect on 6-years survival was evident in diabetic people who have yet survived up to 70 years.

## Introduction

Obesity is a strong predictor of diabetes incidence, while the association between obesity and mortality is more controversial, most of studies showing an inverse relationship between obesity and mortality and a modification effect of age [1]–[8].

Waist circumference positively correlates with abdominal fat mass, providing a simple and reliable assessment of central obesity, which is associated with increased mortality risk. However, prospective data on the predictive role of central obesity on cardiovascular mortality of diabetic patients are very limited [9]. Central obesity and cardiovascular risk factors, such as glycemic control, hyperlipidemia, hypertension and microalbuminuria, are closely interrelated and proper multivariate analyses should be performed to take into account the independent effect of waist circumference. Moreover, emerging evidence has pointed out that natriuretic peptides play a key role in the regulation of body weight and energy metabolism [10]. N-terminal pro-brain natriuretic peptide (NT-proBNP), the inactive molecule resulting from cleavage of BNP pro-hormone, is negatively correlated with obesity and the strongest predictor of CV mortality in diabetic patients [11]–[14]. To our knowledge, however, no prospective study has provided estimates of CV mortality adjusted for NT-proBNP, so that the final effect of central obesity on mortality risk has been probably biased downward. Finally, both in diabetic and non-diabetic patients age has a modification effect on the relationship between BMI and mortality, and a similar pattern could also affect the relationship between central obesity and mortality [3]–[7], [14].

In this study we have assessed the predictive role of central obesity on mortality in people with type 2 diabetes recruited as part of the Casale Monferrato Study, an ongoing population-based study on the epidemiology of diabetes in Italy, after having excluded those who had other chronic diseases impairing short-term survival [15]–[16]. Aims of the present analyses were: 1) to assess the role of central obesity on 6-years mortality risk in diabetic people without other chronic diseases; 2) to define whereas age at the baseline examination was a modifier of the relationship between central obesity and mortality; 3) to examine the effect of NT-proBNP on this associations.

## Methods

The present study was carried out in the second population-based prevalence survey of the Casale Monferrato Study, which identified 3249 people with a previous diagnosis of type 2 diabetes [15]–[16]. As described in detail elsewhere, participants were identified from diabetes clinics and other administrative data sources, obtaining high estimated completeness of ascertainment.

Standardized anthropometric measures were obtained at the local diabetes clinic. Patients with other chronic disease such as heart failure, lung and liver diseases and neoplasm, which could have impaired short-term survival, were excluded from present analyses, giving a final cohort of 2272 subjects. In a random subgroup of 1690/2272 (74.3%) patients who had stored plasma samples, the dosage of NT-proBNP was also performed. With respect to examined patients, they had similar age (67.7±10.4 vs 68.6±10.5 years, p = 0.08), duration of diabetes (10.7±10.3 vs 10.9±10.3 years, p = 0.40), waist circumference (98.0±12.1 vs 90.0±11.9 cm, p = 0.18), HbA1c (7.0±1.8 vs 7.2±1.8), systolic blood pressure (146.0±16.2 vs 145.1±17.2, p = 0.30), diastolic blood pressure (82.5±8.1 vs 82.8±8.2 mmHg, p = 0.19), prevalence of hypertension (90.6% vs 87.8%, p = 0.06) and CVD (22.4% vs 21.1%, p = 0.51), as well as plasma levels of lipids, CRP and uric acid (data not shown).

The study was reviewed and approved by the Ethics Committee of the S.Giovanni Battista Hospital, Turin. All patients gave written informed consent to the study, which was carried out in accordance with the Declaration of Helsinki.

For all patients enrolled, the date of diagnosis was recorded from clinical charts. Weight and height were measured with subjects not wearing shoes. Waist circumference was measured at the midpoint between the lower rib and the iliac crest and central obesity defined as values >102 cm in men and >88 cm in women. Hypertension was defined as systolic blood pressure ≥ 140 mm Hg or diastolic blood pressure ≥ 90 mm Hg or treatment with anti-hypertensive drugs. Smoking habit was classified as never smoked, past smoker if the patient had stopped smoking at least 1 month before the visit, and current smoker. All laboratory determinations were centralized. Venous blood samples for determination of creatinine, triglycerides, total cholesterol, HDL-cholesterol (enzymatic colorimetric method after precipitation with Mn^++^), apoA1, apoB (turbidimetric method, BM/Hitachi 717, BBR, Japan) and hemoglobin A1c (HbA1c) (high-performance liquid chromatography; Daiichi, Menarini, Japan; laboratory reference range, 3.8–5.5%) were collected after overnight fasting. LDL-cholesterol was calculated through the Friedewald’s formula. High-sensitivity (hs)-CRP was measured by immunoturbidimetry (Roche-Diagnostic, coefficient of variation = 0.5%). AER was measured by nephelometry (Behring Nephelometer Analyzer, Behring Institute, Marburg, Germany, coefficient of variation 4%) on single overnight urine collections and categorised as either normoalbuminuria (<20 µg/min) or micro/macroalbuminuria (≥20 µg/min). Serum NT-proBNP levels were measured by a two-site sandwich electrochemiluminescence immunoassay (Elecsys proBNP II, Roche Diagnostic, Mannheim, Germany), using a Modular Analytics Evo analyzer with a E170 module (Roche) as previously described [16]. The intra-assay variation was below 3.0% and total coefficient of variation ranges between 2.2 and 5.8% in low and high ranges of NT-proBNP.

### Statistical Analysis

Variables distributed normally are presented as mean and standard deviation (SD), while variables with skewed distribution (triglycerides, AER, CRP, NT-proBNP) were analyzed after logarithmic transformation and results presented as geometric means and SD. Comparisons were performed using the Student t test and the χ^2^ test as appropriate. Pearson’s correlation was used to assess relationships between waist circumference, age and NT-proBNP.

The relevant time for the analysis was time since diagnosis of diabetes to death or to 31 December 2006, whichever came first, with left truncation for the period of time from onset of diabetes to baseline examination. Information on deaths was obtained from the demographic registries of the town of residence, hospital discharge and autopsy records. Only one patient was lost to follow-up. The underlying causes of death were ascertained and coded according to the ICD-9 classification. Mortality rates were calculated by dividing the number of deaths that occurred during the study period by the whole number of person–years of observation. Excess of risks of death from cardiovascular diseases (ICD-9 codes 390–459) and from all causes were expressed as hazard rate ratios (HRs). Variables-adjusted HRs were calculated using multivariate Cox proportional hazards models. Given the time scale, all models were also adjusted for known duration of diabetes. Models were adjusted for age, sex, diabetes duration (model 1), systolic blood pressure, cardiovascular diseases (CVD), HbA1c, LDL-cholesterol, smoking status, logAER, logCRP, (model 2), and logNT-proBNP (model 3). Subgroup analyses were conducted according to strata defined by median baseline age (<70 years vs ≥70 years). The proportional hazard assumption of explanatory variables, assessed on the basis of Schoenfeld residuals, was confirmed. The likelihood ratio test was used to assess the significance of variables. The *p* value was two-sided; a *p* value<0.05 was considered as being statistically significant. All analyses were performed with STATA (Release 10.0).

## Results

Mean age was 67.9 years, 49.3% were men. People with central obesity were mainly women and had a worst cardiovascular risk profile than those without central obesity (Table 1); indeed, they had higher values of glucose, HbA1c, LDL-cholesterol, apoB, systolic and diastolic blood pressure, and lower values of HDL-cholesterol and apoA1. Prevalence of overweight and obesity were 34.3% and 34.2%. The Pearson correlation coefficient was 0.79 (p<0.0001) between BMI and waist circumference, −0.11 (p<0.001) between age and waist and −0.07 (p = 0.002) between waist circumference and logNT-proBNP, and these results were similar in men and women.

**Table 1:** Characteristics of people with type 2 diabetes of the Casale Monferrato surveys, by central obesity.

	Central obesity		
	Yes (n = 1350)	No (n = 922)	P
**Age** (years)	68.2±10.1	67.5±10.9	0.15
**Men**	42.6%	57.5%	<0.0001
**Duration** (years)	10.8±7.9	10.7±8.1	0.89
**Glucose** (mmol/l)	9.9±3.1	9.2±3.0	<0.0001
**HbA1c** (%)	7.2±1.8	6.8±1.7	<0.0001
**Total colesterol** (mmol/l)	5.56±1.03	5.37±1.03	<0.0001
**LDL-cholesterol** (mmol/l)	3.36±0.88	3.23±0.88	0.0003
**HDL-cholesterol** (mmol/l)	1.40±0.36	1.47±0.44	<0.0001
**Triglycerides** * (mmol/l)	3.64 (2.54)	2.95 (2.01)	<0.0001
**Apo A1** (mg/dl)	155.7±36.5	151.6±42.6	0.02
**Apo B** (mg/dl)	105.2±26.7	98.2±26.4	<0.0001
**Uric acid** (µmol/l)	344.98±113.01	321.19±119.55	<0.0001
**CRP (g/dl)** *	3.6 (1.8–7.4)	1.9 (0.8–4.3)	<0.0001
**NT-proBNP (pg/ml)** *	88.3 (389.8)	87.6 (894.7)	0.91
**Systolic blood pressure** (mmHg)	147.4±16.1	143.41±16.7	<0.0001
**Diastolic blood pressure** (mmHg)	83.8±8	81.2±8.2	<0.0001
**Hypertension**	93.2%	85.0%	<0.0001
**CVD**	22.7%	21.1%	0.37
**Current smokers**	21.0%	11.1%	<0.001
**BMI (Kg/m^2^)**	31.1±4.8	2.0±2.9	<0.0001
**Treatment diet**	13.6%	18.3%	<0.0001
** Oral drugs**	77.2%	69.1%	
** Insulin**	9.2%	12.7%	

Data are shown as mean ± SD.

*geometric means (SD).

Out of the total cohort of 2272 subjects, 520 deaths (221 CV deaths) occurred during a median follow-up of 5.4 years. Risk for both cardiovascular and all-cause mortality were similar in people with and without central obesity (Table 2, model 1).

**Table 2:** Association between central obesity and total mortality and CVD mortality in the Casale Monferrato Study.

	Deaths(n)	Mortality rates/1,000person-years	Model 1 HR*(95% CI)	Model 2 HR**(95% CI)	Model 3 HR***(95% CI)
***Cardiovascular mortality***					
**Central obesity**					
*** All cohort***					
** no**	85	16.8	1.00	1.00	1.00
** yes**	136	19.8	1.14 (0.86–1.52)	1.03 (0.68–1.56)	1.19 (0.74–1.92)
*** Age<70 years***					
** no**	16	5.8	1.00	1.00	1.00
** yes**	44	12.7	2.15 (1.18–3.92)	1.75 (0.75–4.08)	3.52 (1.17–10.57)
*** Age≥70 years***					
** no**	69	37.4	1.00	1.00	1.00
** yes**	92	32.7	0.95 (0.68–1.33)	0.84 (0.51–1.36)	0.80 (0.46–1.40)
***All-cause mortality***					
*** All cohort***					
** no**	197	42.8	1.00	1.00	1.00
** yes**	285	43.4	1.07 (0.88–1.30)	0.97 (0.71–1.29)	0.88 (0.68–1.14)
*** Age<70 years***					
** No**	38	13.8	1.00	1.00	1.00
** Yes**	96	25.5	1.90 (1.28–2.83)	1.38 (0.83–2.20)	1.86 (1.03–3.32)
** Age≥70 years**					
** no**	159	86.1	1.00	1.00	1.00
** yes**	189	67.2	0.90 (0.72–1.13)	0.84 (0.51–1.36)	0.73 (0.51–1.04)

*adjusted for age, sex, diabetes duration.

**adjusted also for systolic blood pressure, HbA1c, LDL-cholesterol, smoking, CVD, logCRP, logAER.

***adjusted also for logNT-proBNP.

As interaction between age and waist circumference was statistically significant (p<0.001), we performed stratified analyses by median age at baseline (<70 vs ≥70 years). Table 2 shows that a strong modification effect of age was evident, with a statistically significant increased risk of both cardiovascular and all-cause mortality in people aged <70 yrs: model 1, HR = 2.15, 95% CI 1.18–3.92 and HR = 1.90, 1.28–2.83, respectively. Risks were reduced by further adjustment for other cardiovascular risk factors (model 2).

NT-proBNP was negatively associated with waist circumference in people aged <70 years and this effect was evident comparing the fully adjusted model 2 with model 3 including also NT-proBNP (HR = 1.75, 0.75–4.08, and HR = 3.52, 1.17–10.57, respectively). In contrast, risk in people aged ≥70 years was similar in people with and without central obesity, irrespective of adjustment for NT-proBNP. Moreover, results were unmodified after further adjustment for BMI, alcohol consumption and treatment with drugs for hypertensions and dyslipidemia. Results were also similar using waist circumference as continuous variable in models 3, which were separately run by sex: cardiovascular mortality, age<70 years, men HR = 1.07 (1.02–1.12); women HR 1.07 (1.00–1.10); age≥70 years, men HR = 0.99 (0.95–1.04), women HR (0.99–0.96–1.03).

## Discussion

This study was conducted to investigate the independent association of central obesity with cardiovascular mortality in a population-based cohort of people with type 2 diabetes who were free of other chronic diseases impairing short-term survival. We found that age was a strong modifier of the relationship between waist circumference and mortality. Indeed, central obesity was associated with a statistically significant 3-fold higher cardiovascular mortality in a 6-years follow-up period of people aged 70 years and lower, whereas no effect was evident in those who had yet survived up to 70 years. This association was independent of classical and novel risk factors. Finally, NT-proBNP was a negative confounder of the association between waist circumference and cardiovascular mortality. The strength of the association between central obesity and CV mortality in people with type 2 diabetes was similar to that estimated in non diabetic people and emphasize the usefulness of this simple measurement in the setting of clinical practice [17]–[20].

Several studies have shown that obese persons have lower circulating natriuretic peptide concentrations than normal weighted persons and a negative linear relationship between BMI, central obesity and NT-proBNP plasma values has been found both in diabetic and non-diabetic people [10]–[14]. This finding has probably biased downward the strength of the association between waist circumference and mortality in previous studies. Interestingly, a significant increase in NT-proBNP values following bariatric surgery has been shown, despite significant improvement in left ventricular diastolic dysfunction [21]. However, to our knowledge no previous study has considered the potential effect of NT-proBNP on the association between central obesity and cardiovascular mortality. The natriuretic system and adiposity are closely linked. Natriuretic peptides have been shown to be important stimuli for lipolysis in humans, which is similar in potency to catecholamines, and their effect is independent of both catecholamines and insulin pathway [22]–[23]. Cardiac natriuretic peptides have emerged as potent metabolic hormones. Experimental studies have shown their role on adipose tissue metabolism and on central control of energy balance [24]. In addition to their impact on lipolysis, NPs are able, via the cGMP/cGK-I pathway, to modulate and inhibit the secretion of adipokines and cytokines, such as interleukin-6, interleukin-8 (IL-6, IL-8) and TNF-α, by adipose tissue, whose increased secretion has been suggested to play a role in the etiology of the chronic state of low-grade inflammation and insulin resistance associated with obesity [25].

Various hypotheses have been suggested as possible explanations of the either negative or weak association between obesity and mortality in the elderly, which might apply to central obesity in diabetic patients [26]. Overweight might confer improved survival during recovery from adverse conditions such as infections or surgical procedures in vulnerable subjects. This protective effect in the elderly might counterbalance an adverse consequence of obesity on survival. As an alternative hypothesis, data derived from observational prospective studies could be affected by survival bias; indeed, the elderly obese people recruited in these surveys might represent the subgroup of healthy obese people who survived up to the baseline examination, thus weakening the association between obesity and mortality.

Our findings are original and have clinical relevance, providing evidence that the measurement of waist circumference is useful to predict risk of death, although limited to diabetic people aged 70 years and lower. Waist circumference cut-off points for the diagnosis of central obesity are a matter of debate [27]–[28]. Although different thresholds are used based on sex and ethnicity, no similar approach has been defined for the elderly. As in developed countries more than two thirds of people with type 2 diabetes are elderly, our findings have a relevant clinical implication, suggesting that recommendations for the elderly need to be individualized.

The present study, relying on well-characterized population-based cohorts of type 2 diabetic people, extends previous observations, assessing simultaneously various factors linked to both central obesity and mortality risk, particularly AER, CRP and NT-proBNP, the strongest predictor of cardiovascular mortality. Other strengths of the study include the analysis of standardized anthropometric measures obtained by trained physicians rather than data based on self-reports, allowing to exclude the effect of misclassification; the population-based study design, allowing to exclude the effect of selection bias; the number of person-years of observation on which data are based allowing to provide quite robust data. By excluding patients who had other chronic diseases at baseline, we minimized the possibility of reverse causation, that is central obesity might be a consequence rather than a cause of mortality. Limitation of this study should also be taken into account. No measurements of either lean body mass or level of physical and cardiovascular fitness were available. Moreover, we could not take into account other potential confounders, such as socioeconomic status, lifestyle and dietary factors apart from alcohol consumption.

In conclusions, data of the Casale Monferrato Study showed that in diabetic people aged 70 years and lower, central obesity was independently associated with a 3-fold increased cardiovascular mortality, independently of the negative effect of NT-proBNP. In contrast, no effect on 6-years survival was evident in diabetic people who have yet survived up to 70 years.

G.B is the guarantor of this work and, as such, had full access to all the data in the study and take responsibility for the integrity of the data and the accuracy of the data analysis.
